# Supplementary material for: Analysis of Polyphenolic Compounds in Water-Based Extracts of Vicia faba L.: A Potential Innovative Source of Nutraceutical Ingredients
Source: Antioxidants (Basel). 2022 Dec 12;11(12):2453. doi: 10.3390/antiox11122453 (PMC9774889; doi:10.3390/antiox11122453)
Supplement: Supplementary file 1 [file antioxidants-11-02453-s001.zip › antioxidants-2044984-supplementary.pdf]

Supplementary Materials:

Table S1. Gastrointestinal digestion solutions.

Salt solution	Stock concentration (mol/L)	SSF (pH 7)		SGF (pH 3)		SIF (pH 7)	
		mL of Stock added to prepare 0.4 L (mL)	Final salt concentration in sample (mmol/L)	mL of Stock added to prepare 0.4 L (mL)	Final salt conc. in sample (mmol/L)	mL of Stock added to prepare 0.4 L (mL)	Final salt conc. in sample (mmol/L)
KCl	0.5	15.1	15.1	6.9	6.9	6.8	6.8
KH_2PO_4	0.5	3.7	1.35	0.9	0.9	0.8	0.8
$NaHCO_3$	1	6.8	13.68	12.5	25	42.5	85
NaCl	2	-	-	11.8	47.2	9.6	38.4
$MgCl_2(H_2O)_6$	0.15	0.5	0.15	0.4	0.12	1.1	0.33
$NH_4(CO_3)_2$	0.5	0.06	0.06	0.5	0.5	-	-

SSF: simulated salivary fluid; SGF: simulated gastric fluid gastric; SIF: simulated intestinal fluid.

Table S2. Bioaccessibility measured in the control capsules

**Samples**	**TPC mg GAE/g ± SD**
Not digested cellulose	0.1±0.00
	**CT**
Digestion Stage	
Intestinal stage	N.D.
Pronase stage	N.D.
Viscozyme L stage	N.D.
Total colonic stage	N.D.

N.D.: not detected; CT: control capsules; TPC: total phenolic compounds.

Table S3. Correlation between DPPH and ABTS tests and data obtained by the Folin-Ciocolteu assay.

**Assay**	**Intestinal Stage**	**Pronase Stage**	**Viscozyme L. Stage**
	R^2	R^2	R^2
DPPH	0.97	0.98	0.97
ABTS	0.97	0.97	0.98

The correlation coefficients were evaluated by using Pearson's method.

Table S4. Antioxidant capacity measured by DPPH and ABTS methods in the control capsules

**Samples**	**DPPH mmol/kg ± SD**	**ABTS mmol/kg ± SD**
Not digested cellulose	**0.2 ± 0.0**	0.2 ± 0.0
	**CT**	**CT**
Digestion Stage		
Intestinal stage	0.1 ± 0.0	0.1 ± 0.0
Pronase stage	N.D.	N.D.
Viscozyme L stage	N.D.	N.D.
Total colonic stage	N.D.	N.D.

N.D.: not detected; CT: control capsules.
